# Influences on uptake of reproductive health services in Nsangi community of Uganda and their implications for cervical cancer screening

**DOI:** 10.1186/1742-4755-4-4

**Published:** 2007-06-26

**Authors:** Twaha Mutyaba, Elisabeth Faxelid, Florence Mirembe, Elisabete Weiderpass

**Affiliations:** 1 Department of Obstetrics and Gynaecology, Makerere University Medical School, P.O.Box 7072, Kampala, Uganda; 2 Department of Medical Epidemiology and Biostatistics, Karolinska Institutet, Stockholm, Sweden; 3 Department of Public Health Sciences, Division of International Health Care and Research, Karolinska Institutet, Stockholm, Sweden; 4 Department of Etiological Research, The Cancer Registry of Norway, Oslo, Norway; 5 Samfundet Folkhälsan, Helsinki, Finland

## Abstract

**Background:**

Cervical cancer is the most common female cancer in Uganda. Over 80% of women diagnosed or referred with cervical cancer in Mulago national referral and teaching hospital have advanced disease. Plans are underway for systematic screening programmes based on visual inspection, as Pap smear screening is not feasible for this low resource country. Effectiveness of population screening programmes requires high uptake and for cervical cancer, minimal loss to follow up. Uganda has poor indicators of reproductive health (RH) services uptake; 10% postnatal care attendance, 23% contraceptive prevalence, and 38% skilled attendance at delivery. For antenatal attendance, attendance to one visit is 90%, but less than 50% for completion of care, i.e. three or more visits.

**Methods:**

We conducted a qualitative study using eight focus group discussions with a total of 82 participants (16 men, 46 women and 20 health workers). We aimed to better understand factors that influence usage of available reproductive health care services and how they would relate to cervical cancer screening, as well as identify feasible interventions to improve cervical cancer screening uptake.

**Results:**

Barriers identified after framework analysis included ignorance about cervical cancer, cultural constructs/beliefs about the illness, economic factors, domestic gender power relations, alternative authoritative sources of reproductive health knowledge, and unfriendly health care services. We discuss how these findings may inform future planned screening programmes in the Ugandan context.

**Conclusion:**

Knowledge about cervical cancer among Ugandan women is very low. For an effective cervical cancer-screening programme, awareness about cervical cancer needs to be increased. Health planners need to note the power of the various authoritative sources of reproductive health knowledge such as paternal aunts (*Sengas*) and involve them in the awareness campaign. Cultural and economic issues dictate the perceived reluctance by men to participate in women's reproductive health issues; men in this community are, however, potential willing partners if appropriately informed. Health planners should address the loss of confidence in current health care units, as well as consider use of other cervical cancer screening delivery systems such as mobile clinics/camps.

## Background

Uganda is a poor country, with a mainly rural population mostly engaged in subsistence farming. Literacy is estimated at about 64 % for men and 47 % for women. Uptake of reproductive health (RH) services is poor, with 23 % contraceptive prevalence, and 38 % skilled attendance at delivery, ten percent postnatal care attendance, and maternal mortality ratio of 505 per 100, 000 live births. Attendance to one antenatal care visit is 90%, but less than 50% complete three or more antenatal visits, as advised by WHO [1]. The reasons for high antenatal attendance at least for one visit and the low usage of other services are unknown, and may be due to the fact that pregnant women are given a visit card in the first antenatal consultation, which may facilitate their access to public health units in case of emergencies.

Cervical cancer is the most common female cancer in sub Saharan Africa and accounts for the highest female cancer related mortality. In East Africa, estimated age-standardized incidence rates for cervical cancer are 42.7 and mortality estimated at 34.6 per 100,000 women-years. Cervical cancer is the commonest female cancer in Uganda with an estimated age standardized incidence rate of 40 per 100,000 [2-4]. Most women diagnosed or referred with cervical cancer in Mulago, the national referral University hospital, have advanced disease. Commonly, women with cervical cancer present to the clinics with intermenstrual bleeding, contact bleeding or offensive vaginal discharge [5,6]. There is a relative paucity of research focused on cervical cancer from Uganda. In Uganda, as in most other sub-Saharan countries, there is no organised screening programs for cervical cancer, and opportunistic screening, i.e. screening services offered to women arriving to a health care service for any other reason, is available only in a few health care units and is rarely used [7,8].

Whereas Pap smear screening programs have been very successful in reducing incidence of cervical cancer in high-income countries [9-11], they are costly and logistically difficult to be implemented in low-income countries. Lack of screening certainly accounts for at least part of the high incidence and mortality rates of cervical cancer. Screening by visual inspection [12-18] has been proposed as an alternative method for low income countries. In these countries, clients surmount enormous barriers to reach a health care facility, and offering immediate treatment of suspicious cervical lesions after screening by visual inspection could optimize compliance s [14,19,20].

Based on studies carried out in countries where organized screening is available, it is known that screening uptake can be influenced by cultural beliefs, the social position of women, characteristics of the health care system, the physician's attitudes towards screening and women's comprehension of the screening process. Embarrassments about undergoing a gynecological examination, fear of the procedure or belief that little can be done to prevent cancer are other factors that might decrease screening participation. Lower socio-economic background, lack of health insurance and low literacy also compromise participation in screening. Attending cervical cancer screening may have a negative connotation or stigma when it is combined with a gynecological examination and treatment for reproductive tract infections. The gender of health care professionals and limited time that they allocate to patient education may negatively influence screening participation as well [21]. Other influences that may influence participation in screening in particular in low resource countries are gender imbalances [22-24] and whether illness is perceived as traditional or modern [22,25-28]. Adequate knowledge about cervical cancer influences early detection [29] and treatment seeking pattern [30].

The aim of this study was to explore, in a semi-rural community of Uganda, factors that influence uptake of cervical cancer screening and explain the low usage of available reproductive health services. In Uganda, there is a national policy of integrated reproductive health service delivery. Non-use of the other services i.e. antenatal care, postnatal care, family planning and gynecological services would impact the usage of cervical cancer screening services since they are vital entry points in the health care system for information, education and screening of women.

## Methods

Focus group discussions (FGDs) were held with community members, women attending a postnatal/family planning clinic and nurses/midwives. Discussions with community members were conducted in Nsangi, a semi-rural community about ten kilometres from Kampala, the capital city of Uganda. The FGDs with patients and nurses/midwives were held in Mulago hospital postnatal/family planning clinics. Mulago is the national referral hospital for Uganda and is the teaching hospital for Makerere University medical school. It is the largest and busiest hospital in Uganda.

### Participants

The eight FGDs had 8–15 participants each and comprised of the following categories: two groups of married women in Nsangi, two groups of married men in the same community, two groups of women attending the postnatal/family-planning clinic at Mulago hospital and two groups of nurses/midwives in the family planning/postnatal clinic also at Mulago hospital. The age range of participants was 28 to 63 years. The majority of participants belonged to the Baganda ethnic group, the largest tribe, representing about 20% of the population of Uganda. The participants from Nsangi community were mainly subsistence farmers, which is the commonest occupation in Uganda. We selected women who came to the postnatal clinic as representatives of the very few women (10%) who come for that service in Uganda [1]. Nurses form the bulk of the Ugandan health work force and are therefore familiar with the day-to-day problems of health service delivery. The nurse in charge of the postnatal/family-planning clinic invited women who sought family planning services and routine postnatal care. All participants gave verbal informed consent. No one declined participation. We had ethical clearance from Makerere University research committee and the National Council for Science and Technology.

### Process

All discussions were conducted according to a FGD guide, developed in English and translated into Luganda, the main local language. The guide contained an introduction, purpose of the meeting, rules during the discussions e.g. confidentiality and time, encouragement of open all-inclusive discussion, non-disclosure of personal information and consent to tape record the discussion and the topics for discussion. These included knowledge about cervical cancer, cultural beliefs and knowledge sources, definitions and understanding of the disease, socio economic factors, domestic gender power relationships and service delivery factors. Case vignettes depicting symptoms of cervical cancer were used during the discussions with the non-medical participants to determine knowledge of the disease, whether there were cultural equivalents with similar symptoms, and health seeking behaviour in case of such symptoms.

The discussions lasted 60 to 90 minutes and were all tape-recorded. A moderator guided the discussions. Each group chose a "leader" among the participants who worked with the moderator to guide the interview by allocating time and opportunity to each speaker, to encourage an all-inclusive atmosphere. The use of this was contextual. In Uganda since 1986, there is a practice of holding village meetings called Local Councils, to deliberate on issues in the communities. They are quite similar to focus group discussions. We took advantage of this to achieve maximum contribution by the participants.

Another investigator attended the discussion as an observer and took notes. At the end of each discussion, the moderator and observer confirmed that all information was captured. The observer would give the moderator feedback on the conduct of the discussion. We deemed FGDs to be the best method to achieve our objectives as has been noted by other researchers [31,32].

We found out early in the investigation that the community participants were largely unaware about cervical cancer. We therefore used other reproductive health services i.e. antenatal care, delivery and family planning as proxy indicators to understand their views on usage. Emerging themes that developed from the discussions were further explored. We raised the issue of male negligence on women's reproductive health issues with the men and played back some taped discussions we had had with the women. We did the same with the nurses, playing back the tapes with the allegations of the mal-treatment of women clients. This was done to convince them that the issues we raised were genuine concerns and not just our imagination.

### Analysis

We conducted framework analysis as described by Ritchie and Spencer [33]. Analysis was continuous from the start of the data collection. The taped discussions were transcribed within three days. We read and re-read the transcripts and observation notes to familiarize ourselves with the range of issues. We then developed a thematic framework from the a priori themes and emergent themes. This was then applied to the data to sort the data according to the themes (these were titled as presented in the findings). We then sought to explain the findings from a Ugandan context and interpret the implications to any future planned cervical cancer prevention programme.

## Results

Findings are presented according to the themes identified in the analysis. In some of the quotes, terms in Luganda are used; meanings are given in appendix 1. The a priori themes we started with were confirmed during FGDs as important in influencing uptake of services. Emergent themes were male partner influence on women's health-seeking behaviour and authoritative sources of reproductive health knowledge.

From the FGDs with community members, cervical cancer did not feature prominently among their health problems. We asked participants to identify reproductive health problems especially concerning women. Women mentioned: pregnancy related complications, diseases of the fallopian tubes ("*enseke"*), menstrual problems, tumours of the uterus, cancer related to use of the pill and infertility. Men mentioned: deaths during childbirth, tubal diseases, syphilis (described as vaginal sores and discharge), "*Kyaalaalo*"(chronic vaginal bleeding) and attributed to contraceptive usage, "*Nabbuguma*" a condition described as 'heat in the uterus', which causes recurrent intrauterine foetal deaths or abortions, and is attributed to alcohol consumption, smoking, or heredity. Many men also mentioned unplanned pregnancy as a problem.

### Knowledge on cervical cancer

Non-medical participants were asked whether they had ever heard of this cancer, what they knew about it, what they knew caused it, what they called it in local terms and where they would go for treatment. In the community groups, most participants did not know about cancer of the "mouth of the womb". Only one woman had heard about it on radio but did not have details. The majority of the women from the postnatal clinic (PNC) had never heard about cervical cancer. They could not describe the symptoms but mentioned use of contraceptives and having sex during menstrual period as causes. *"My mother told us that if you have sex during menstruation, you could get cancer of the womb" *(Woman in PNC). *"We hear that cancer is due to contraceptives yet you are encouraging us to use them"*- (Woman in PNC). On using a case vignette depicting cervical cancer, many related the symptoms to "*kabotongo*"(syphilis).

Ignorance about the cancer could partly be due to health workers' practices. They were said to rarely inform their clients about the diagnosis.

*"Usually the health workers do not tell you what you are suffering from. We go back home with treatment but without knowing the disease. Even the way they write one cannot read and understand"*- (Woman in community)

Among the health workers, the following questions were posed for discussion. What is cervical cancer? What causes cervical cancer? Who is at risk for it? How do I know it is the one? Is it a public health problem or not? What does the nurse/midwife do when she suspects a client to have cervical cancer? Is it treatable or not and what treatment options are available?

All the nurses knew about cervical cancer and deemed it a public health problem. Many had good knowledge about it but some had some inaccurate information regarding various aspects of the cancer. *"I would tell the clients about symptoms especially bleeding after sex and other abnormal vaginal bleeding. One should go for a pap smear if above 35 years of age"*- (Nurse). *"One of the causes is use of local herbs especially in pregnancy"*- (Nurse).*"It is a deadly disease. Early sex and frequent abortions are the cause. It is difficult to diagnose that is why most people come with advance disease"*- (Nurse).

### Cultural constructs about cervical cancer (and related genital diseases) and authoritative sources of knowledge

Some discussants perceived the illness a "traditional" disease. When symptoms were described many related them to a traditional condition called *"kikulukuto" "That is kikulukuto. It is when a woman does not stop menstruating. It is related to these family planning things they use"- *(Man in community). Most women cited their *"Senga" *(paternal aunt), mother, elderly women and peers as the authoritative source of knowledge about the illness and other reproductive health issues. Surprisingly, health workers were distant as authoritative sources of knowledge.

*"If my mother told me that the illness is traditional, I would believe her and seek treatment at a traditional healer even if a doctor said it is not so" *(Woman in PNC)

We described a woman with symptoms of advanced cancer of cervix who had delayed to come to hospital and asked the women in the discussion group for explanations. One discussant responded, *"She thought it was a traditional disease. Women in the villages start with local herbs. Only when they fail to get better do they come to hospital"- *(Woman in PNC)

### Economic factors and male partner influences

Money was a prominent factor in influencing health-seeking behaviour *"We have many problems e.g. school fees, food etc. One has to prioritise, so when you get an illness, you first buy some medicines. If the disease grows, then you go to hospital. We hear of so many medicines on radio which relieve pain, so we use them first"*- (Woman in community).

Though medical care in government units is supposed to be free, there are informal charges and even bribery in the government units that keep the women away. *"When you do not offer money in government hospitals you do not get care. They should tell us clearly how much and where to pay"*-(Woman in community).

These were lent credence by the following testimony of a nurse working in a government health unit *"One comes to work expecting to go back with something. The clients who buy something e.g. gloves from us are better attended to"*- (Nurse).

At domestic level, the men control the purse. *"Our husbands are the ones who have the money. Most men do not want to give you money. They are just like that. If both of you get a private disease, he will go and get treated and leave you to suffer"*-(Woman in PNC)

There is reticence by the women to inform their partners about reproductive health issues. Culturally, men in this ethnic group are not expected to get involved in reproductive health problems of their women and there is a lack of dialogue at home regarding reproductive health issues. *"I do not tell my partner all illnesses especially those involving the private parts. He might reject me and get another woman"*- (Woman in PNC). *"Men! Even if they brought the disease, they would accuse you of having brought it. If I need money for a private illness, I tell him I have a different illness"*- (Woman in PNC)

Men's reasons for not getting involved were; cultural norms regarding female reproductive health problems, fear of having to pay the health workers if they went with the women to hospital, and not knowing, as the women do not tell them of their problems.*"In Baganda culture, when my wife gets reproductive health problems, it is my sister or her Senga to accompany her to hospital. The man does not have to accompany her unless specifically requested by the doctors for joint treatment as in syphilis"*- (Man in community).

*"When you go with the woman and the health workers see you, they know that there is money available. That is one of the reasons why we do not go with them to hospital"- *(Man in community).

*"Women most of the times tend to hide diseases of private parts from us. They fear loss of their marriages, therefore seek treatment on their own"*- (Man in community).

Uganda is a patriarchal society. Even when a woman is employed, she will expect the partner to meet the costs for health care. Most women were of the view that men were not meeting this role. They suggested that men needed to be educated about women's reproductive problems and needs. In contrast to the women in the community who put all financial expectations on the male partner, women in the postnatal clinic seemed to be more empowered. A question was put to them on how they managed to come to hospital for postnatal care since majority of women do not come back. *"My husband facilitated me. I told him and showed him the discharge form with the appointment date and he gave me the money"*- (Woman in PNC)

*"I plan. I keep aside some money. When he does not have, I use that to go to hospital"*- (Woman in PNC)

### Health services factors

Participants voiced concerns about public (Government) health units. They were said to be under-equipped, understaffed and lacked medicines. **"***Facilities in government hospitals are lacking. They ask you for everything, gloves, polythene sheets and medicines. I would not mind the lack of beds but there is no cleanliness" *(Woman in community).

The nurses, especially female nurses, were said to be rude, uncaring and sometimes cruel to patients. "*The nurses are very rude. The women tell us that they would rather be attended to by male health workers*" (Man in community). *"There should be more male health workers. The female nurses are very rude" *(Woman in community). *"Because of low pay, some nurses work during the day and then come for night duty. They are too tired and so steal some sleep and ignore the patients or become rude"- *(Nurse)*. "You see, some nurses change and cease to be rude to patients when they work with projects which pay well"*- (Nurse).

In Mulago, the national referral hospital, conditions were said to be very unhygienic especially in the labour ward. *"I went to see my sister who had delivered in Mulago hospital. They were put six women on one mattress on the floor. It had blood all over. I got scared and would not recommend anyone to go there*" (Woman in community).

Waiting time is too long in many units with no privacy especially at Mulago where many students converge on one patient.*"There is no privacy. There may be more than four health workers all examining your private parts. Often, many students are on the side watching" *(Woman in community).

We raised the concerns of women during the discussion with nurses in the postnatal/family planning clinic. We asked them why they are sometimes unfriendly to the women as the testimonies on tapes depicted. We also asked the nurses to discuss the constraints met during the course of their duties especially taking pap smears. They cited lack of supplies, being overworked yet underpaid. "*We lack equipment to use. We run out of slides to do pap smears*" (Nurse). "*We are understaffed. You lose your temper when you have to do so many things at a go*" (Nurse).*"Take the example of labour ward. You can face a line of fifty mothers. Nobody appreciates and when things go wrong you are blamed instead. Possibly that is why you transfer the anger to the patients" *(Nurse).

## Discussion

### Knowledge on cervical cancer and authoritative cultural knowledge

Knowledge was low among the non-medical participants and inaccurate among the nurses. Though cervical cancer has been the commonest cancer of women even before the HIV epidemic, it has been a neglected problem. There are no systematic prevention programs or population awareness campaigns. Furthermore, cervical cancer is seen not as dramatic as HIV/AIDS, malaria, tuberculosis, maternal deaths, infant mortality and other infectious childhood illnesses. The low awareness levels about cervical cancer may be partially explained by the relatively rareness of the condition, and by the fact that its clinical symptoms may mimic other gynaecological conditions, such as infections. Participants from the community intimated that regarding reproductive health issues, they would consult aunties, mothers and peers. Health workers were not included as authoritative sources of knowledge. Ample anthropological literature is available to explain the construction of authoritative knowledge in societies and medical pluralism [27,34-36]. The *"Sengas" *have much power in the construction of this knowledge among the Baganda ethnic group. Such authoritative sources of cultural knowledge have great influence on women's preference for traditional healers as has been found in other studies [34,37-41].

When the services are available, the women should be educated about the cancer in such a way that they recognize the need for getting screened. In doing this, it would be advisable to somehow involve the traditional health system and the existing authoritative sources of reproductive health knowledge e.g. the *"Sengas"*. Health care planners will have to take into account the fact that the cancer is caused by a sexually transmitted agent. Fortunately Uganda has vast experience with HIV/AIDS and has moved towards eliminating the stigma associated with sexually transmitted infections [42,43].

The lack of depth on knowledge of cervical cancer by the nurses can be explained by the nurses' curriculum. Until recently, cervical cancer prevention issues have been the concerns of physicians. No nurse mentioned during the discussions that cervical cancer is actually caused by a sexually transmitted agent. Two new options are now available in the prevention of cervical cancer; vaccination against human papillomavirus (HPV) and HPV testing technologies, which, whenever it becomes affordable, could be an initial screening tool for high risk HPV infections [44,45].

In the proposed diagnosis and treat strategies [16,46] there will be need to integrate cervical cancer prevention issues in the nurses' training curriculum. Nurses will form the backbone for informing the population, as they are the first port of entry into the health system. It remains to be seen what approach is best for cervical cancer control for the country.

### Economic factors and male partner influences

Uganda is still a patriarchal society. Whereas the men are the breadwinners especially in rural areas, it is the women who know the priorities within the homes. This can be detrimental to women's health as they have more pressing issues within the homes on which to spend the little money available rather than on their own health needs. In this poor country, user fees and unofficial charges in the government health units combined with unfavourable domestic gender power relations will keep the women away as has been found in other studies [23,24,47-52]. More investment by government in health services may not be possible at the moment. Many of the participants were not completely against user fees. The problem is the unofficial fees and bribes. There is need to regulate and control user fees and show an improvement in the services on offer e.g. availability of medicines and other supplies like gloves.

Many reproductive health programs recommend encouragement of male participation in reproductive health issues [19,53]. Few studies have explained the reasons for men's reluctance to get involved in women reproductive issues. Our study identified some of the reasons for this situation. Men are not fully to blame for non-involvement in women reproductive issues. Cultural factors, economic factors and reticence on part of the women to inform them about the problems underpin the perceived reluctance of the men. They are potentially willing partners if appropriately informed.

### Health services factors

The quality of health services impacts greatly on usage as has been found in family planning programs [54-56]. Participants in our study had negative experiences of the performance – especially of the public health units. The ministry of health in Uganda encourages integration of health service delivery. For cervical cancer screening, information campaigns could start during antenatal care, which is highly attended for at least for one visit. The opportunistic screening could then be applied in the postnatal and family planning services. Measures should be taken to increase attendance of these services in general, and for screening in particular. One unique finding of this study was that women did not cite the sex of the health workers as a barrier to their use of the health services. With no confidence in stationary clinics, other delivery systems like specific mobile clinics/screening camps need to be considered. They are targeted to cervical cancer screening and field colposcopes have been developed (DIVLABS, India). Cryotherapy, the mainstay of treatment of early cervical lesions could be done at the same visit. The stationary clinics could then be used mainly for women with lesions that require more elaborate management. These systems of service delivery have been effectively used in a low resource setting in India [14,57] but are yet to be tested in the Ugandan setting.

## Conclusion

Knowledge about cervical cancer is very low in our setting. For an effective cervical cancer-screening program awareness about cervical cancer needs to be increased. Health care planners need to note the power of the various authoritative sources of reproductive health knowledge such as *"Sengas" *and involve them in the awareness campaign. Cultural and economic issues dictate the perceived reluctance by men to participate in women's reproductive health issues; otherwise they are potential willing partners if appropriately informed. Health care planners should address the loss of confidence in current health care units where other reproductive health care services such as antenatal care, postnatal care and family planning are provided, since they are crucial entry points for a cervical cancer screening service. Alternatively they may consider use of other delivery systems like mobile clinics/camps.

## Competing interests

The authors declare no competing interests. The funding agency had no influence in the design, accomplishment or interpretation of the results presented here.

## Authors' contributions

TM, EW and FM were responsible for the concept, full proposal development and getting approval from the ethics committees. TM collected the data and carried out the analysis. TM, EW, EF wrote the manuscript and EW and EF were responsible for the preparation of the manuscript for submission. All authors read and approved the final manuscript.

## Appendix 1. Terminologies

***Kabotongo ***– Syphilis. In the local population, various symptoms, which include skin rashes, genital sores and discharge from the vagina, are often wrongly attributed to syphilis.

***Kikulukuto ***– This is a condition when a woman gets either prolonged or frequent vaginal bleeding that may be menstrual or not.

***Enseke ***– Refers to the fallopian tubes. It is common for women with lower abdominal pain to believe that they are suffering from disease in their fallopian tubes.

***Kyaalaalo ***– This has symptoms similar to Kikulukuto but may have pus discharge from the vagina in addition.

***Nabbuguma ***– This is a condition when a woman gets recurrent pregnancy loss either abortion or intrauterine foetal deaths. It is said she has too much heat in her womb. This may be attributable to alcohol consumption, smoking, witchcraft or hereditary factors.

***Siliimu ***– This was a coined name from the English word to slim (Lose weight). The most striking feature of AIDS victims in the early 1980s was weight loss.

***Sengas ***– This refers to the paternal aunt. Among the Baganda, she is responsible for educating the young females (Nieces) on reproductive and sexual issues. She is an authoritative source of cultural knowledge especially, reproductive, sexual and marriage relations.

## Appendix 2. Focus Group Guide for Study on Influences on Uptake of Reproductive Health Services

1. Introduction

• Researchers introduce themselves to the participants

• Participants introduce themselves.

• Explain purpose of meeting.

2. Rules of the meeting/discussion

• Time keeping

• Mode of discussion

• Who selects contributor

• Inclusion of all

• Avoid airing personal issues or information

• Assurance of confidentiality

• Consent to tape record the discussion

• Feed back- summary of deliberations and consensus on content.

**3.Choosing leader from participants to help researchers in moderating discussio**ns

• Consensual choice, majority decision

• Researchers explain to the chosen leader the roles.

4.Issues for discussion (Depending on composition of group)

• Common reproductive health problems they encounter.

• Explanations/Understanding of these illnesses (Cultural knowledge) -construction of, authoritative sources.

• Non-medical participants- (use case- vignette), probe for specific knowledge about cancer of the cervix- local name for the illness with such symptoms, and health seeking behaviour in case of such symptoms.

• For nurses – discuss risk factors, presentation, diagnosis, treatment and prevention

• Decision making in the home and who meets the costs for reproductive health issues.

• Problems faced in getting care for reproductive health problems.

• Male participation in women's reproductive health issues.

• Performance of health units and expectations.

• Health workers- problems faced in service delivery.
